# Editorial Note: Attribute encryption access control method of high dimensional medical data based on fuzzy algorithm

**DOI:** 10.1371/journal.pone.0355451

**Published:** 2026-08-12

**Authors:** The PLOS One Editors

The *PLOS One* Editors issue this Editorial Note to inform readers that works cited in this article [1] as References 10, 11, and 30 were retracted before the article’s publication date. PLOS considers that these references are not crucial in supporting the research or conclusions reported in [1], but the following sentences are no longer supported:

- 1. Introduction, paragraph 1 sentence 30: “Simultaneously, it is imperative to safeguard sensitive data from unauthorized access.”
- 2. Related work, paragraph 1 sentences 4–9: “Boomija et al. proposed a medical data security access control method based on role-based partially homomorphic encryption user policies. This method indicated that access control policies in cloud environments were more flexible, and attackers could easily collect sensitive data by abusing the access policies of other users. However, the secure access data encryption model developed in cloud environments solves this problem. This model was implemented in Eclipse IDE and AWS Toolkit for Eclipse, and deployed in the Amazon Elastic Beanstalk (EB) environment. It was specifically designed to protect patients’ electronic health details. Although Amazon Elastic Beanstalk provides certain security features, the security of the model also depended on developers’ correct configuration and use of these features.”
- 7.1 Trust calculation of access behavior based on dynamic grouping, paragraph 2 sentence 5: “The key characteristics of entity trust include data confidentiality, protection data completeness, and credibility.”
